# Origin and fate of pseudogenes in Hemiascomycetes: a comparative analysis

**DOI:** 10.1186/1471-2164-11-260

**Published:** 2010-04-22

**Authors:** Ingrid Lafontaine, Bernard Dujon

**Affiliations:** 1 Unité de Génétique Moléculaire des Levures, Institut Pasteur, Paris, France; 2 Unité de Formation et de Recherche 927, Université Pierre et Marie Curie, Paris, France; 3 Unité de Recherche Associée 2171, Centre National de la Recherche Scientifique, France

## Abstract

**Background:**

Pseudogenes are ubiquitous genetic elements that derive from functional genes after mutational inactivation. Characterization of pseudogenes is important to understand genome dynamics and evolution, and its significance increases when several genomes of related organisms can be compared. Among yeasts, only the genome of the *S. cerevisiae *reference strain has been analyzed so far for pseudogenes.

**Results:**

We present here the first comparative analysis of pseudogenes within the fully sequenced and annotated genomes of eight yeast species, spanning the entire phylogenetic range of Hemiascomycetes. A total of 871 pseudogenes were found, out of which mutational degradation patterns and consequences on the genetic repertoire of each species could be identified. We found that most pseudogenes in yeasts originate from mutational degradation of gene copies formed after species-specific duplications but duplications of pseudogenes themselves are also encountered. In all yeasts, except in *Y. lipolytica*, pseudogenes tend to cluster in subtelomeric regions where they can outnumber the number of functional genes from 3 to 16 times. Pseudogenes are generally not conserved between the yeast species studied (except in two cases), consistent with their large evolutionary distances, but tend to be conserved among *S. cerevisiae *strains. Reiterated pseudogenization of some genes is often observed in different lineages and may affect functions essential in *S. cerevisiae*, which are, therefore, lost in other species. Although a variety of functions are affected by pseudogenization, there is a bias towards functions involved in the adaptation of the yeasts to their environment, and towards genes of unknown functions.

**Conclusions:**

Our work illustrates for the first time the formation of pseudogenes in different branches of hemiascomycetous yeasts, showing their limited conservation and how they testify for the adaptation of the yeasts functional repertoires.

## Background

Since their original discovery [1], pseudogenes have been found in all studied genomes so far, within the three kingdoms of life [2-4]. Their proportions vary greatly, however, from one organism to another, depending on lifestyle (free-living or association) and on genome properties (rates of duplication, mutation, deletion, and retro-transposition). Pseudogenes correspond to *ca*. 3% of the gene repertoire of *Drosophila melanogaster*, while there are approximately as many pseudogenes as functional genes in the human genome (http://pseudogenes.org). Pseudogenes are often species-specific and, within small genomes, tend to accumulate in chromosomal regions such as subtelomeres or heterochromatin, minimizing possible deleterious effects [5-7].

The historical definition of a pseudogene is a DNA sequence that looks like an active gene but has lost its ability to code for a functional product, due to more or less extensive mutational disablements. Pseudogene formation is frequently observed in pathogenic organisms undergoing reductive evolution while benefiting from host functions [8-11]. Pseudogenes can also correspond to the non-functionalization of a duplicated gene copy [12,13] that originated either from DNA duplication or from retro-transposition [14,15]. Sequence degradation may range from a single disabling mutation, such as a frameshift or an in-frame stop codon in a protein-coding gene, to extensive changes including numerous insertions and deletions [7,16].

In all genomes, most pseudogenes are likely to disappear with time by the accumulation of successive mutations [9]. Those with limited alterations, however, may be repaired by reverse mutations, gene conversion or may be reactivated by translational recoding events [17,18]. Some pseudogenes can also acquire a new functional role, such as the control of another gene expression, or the generation of genetic diversity (see [19] for a review), and give rise to new genes, such as the human *XIST *non-coding RNA gene, which evolved from a pseudogene of a protein-coding sequence [20].

In Fungi, only pseudogenes of particular genes or pathways of interest have been described [21-24]. In *Saccharomyces cerevisiae*, two systematic analyses performed by different approaches [6,7] both concluded to the paucity of pseudogenes of anciently protein-coding sequences in the yeast genome. In order to determine the mechanisms of pseudogene formation across several related species, their age and the functions affected by pseudogenization, we performed the first systematic comparative analysis of the pseudogene repertoire in a set of eight hemiascomycetous yeasts spanning a large evolutionary range, similar or larger than the phylum of Chordates [25,26]. We show that these genomes also contain a limited number of pseudogenes, independently of their global level of gene redundancy. Most pseudogenes originate from duplicated gene copies resulting from previous DNA duplication events, but a few could correspond to retro-processed sequences. Some pseudogenes are formed from single-copy gene and hence correspond to a functional loss in the yeast species. The relative paucity of pseudogenes suggests that functionally inactive genes are rapidly eliminated in hemiascomycetous genomes because their sequence diverges rapidly or because they are successively truncated, or entirely deleted.

## Methods

### Genomes and protein sequences

Sequences and annotations were taken from the Génolevures database (http://www.genolevures.org) for *Candida glabrata*, *Zygosaccharomyces rouxii*, *Kluyveromyces lactis*, *Kluyveromyces thermotolerans*, *Saccharomyces kluyveri*, *Debaryomyces hansenii *and *Yarrowia lipolytica*, and from the *Saccharomyces *Genome Database (http://www.yeastgenome.org/) for *Saccharomyces cerevisiae*. All these sequenced strains are haploids. Note that the annotation files already contained 457 protein-coding pseudogenes. The protein databank used for comparison contains the translational products of the 44,174 annotated CDS from all eight studied genomes (http://www.genolevures.org/yeastgenomes.html). We used the protein family classification constructed by the Génolevures consortium [27].

Whole genome shotgun sequences of *S. cerevisiae *haploid strains AWRI1631, JAY291, M22, RM11-1a, YJM789, YPS163 and EC1118 were retrieved at EMBL (EMBL: ABSV00000000, ACFL01000000, ABPC00000000, AAEG01000000, AAFW00000000 and ABPD00000000, respectively), as well as the genomic scaffolds of the strain EC1118 (EMBL: FN393058-FN393060, FN393062-FN393087, FN394216, and FN394217).

### Identification of the pseudogenes

We developed a set of automatic procedures to obtain an exhaustive list of the potential pseudogenes of anciently protein-coding genes in a given genome. Each genome sequence is given to FASTY [28] and translated into the 6 frames for detection of amino acid similarity against the translational products of the 44,174 annotated CDS from the studied genomes (no pseudogenes from full protein sequences encoded in the nuclear genomes could be retrieved outside of the hemiascomycetes in the Uniprot database).

The matches selected have at least 25% identity and are considered statistically significant if their estimated Z-score [29] is greater or equal to 200 (corresponding to E-values lower than 10^-6^). This cut-off was chosen after examining the distribution of the Z-scores for all obtained results. When several matches overlap, the similarity region is delimited from the left-most to the right-most aligned sequences.

### Protein domain conservation

All matches similar to a protein of unknown function were queried against the PFAM database [30] of conserved domains with the HMMER algorithm[31].

### Identification of the pseudogenes in S. cerevisiae strains

The regions corresponding to the pseudogenes in the *S. cerevisiae *reference strain S288C were retrieved in the whole genome shotgun sequences of the strains AWRI1631, EC1118, JAY291, M22, RM11-1a, YJM789 and YPS163 by a FASTA search at the nucleotide level. These identified regions were then queried against the protein bestmatches of the corresponding pseudogenes in S288C with FASTY. We concluded to the presence of a pseudogene in the considered strain if evidence for coding sequence degradation is proposed in the FASTY alignment (see criteria 3 in section below). The pseudogenes present in strains M22 and YPS163 but not in S288C were identified based on the work by [32].

### Selection criteria

Pseudogenes are defined from the similarity regions using the following criteria: 1) no overlap or partial overlap with an already annotated functional genetic element on the same DNA strand; 2) possible overlap of less than 100 nucleotides with an already annotated functional genetic element on the opposite DNA strand; 3) evidence for coding sequence degradation: at least one in-frame stop codon, one frameshift mutation, or a truncation of at least 30% relatively to the cognate CDS (truncated pseudogenes smaller than 120 nucleotides are ignored, unless their cognate CDS is itself smaller than 120 nucleotides).

Each selected potential pseudogene is aligned to its bestmatch with GENEWISE [33] to predict the corresponding coding sequence. Fifteen candidates were discarded after this step because GENEWISE predicted an intact CDS while FASTY introduced a frameshift mutation in the alignment.

The pseudogenes identified in this study are available at the Génolevures website (http://www.genolevures.org/). The algorithm for pseudogene detection was written for our own analysis, considering that annotations of the genomes studied are of high quality. It is available upon request to the authors.

### Pseudogene nomenclature

Each detected pseudogene is numbered serially from left to right of the chromosome, based on the nomenclature proposed in [34]. The name indicates the species (four letters), the project or strain number (one numeral), the chromosome (one letter), and the pseudogene nature of the sequence ("p") followed by the serial number (for example, CAGL0Ap1).

### Sequence divergence analysis

We assumed that substitutions at the third codon position of a protein coding sequence (or an ancient protein coding sequence) evolve according to a neutral molecular clock. Estimations of the evolutionary distances by several substitution models (JC69, F84 and HKY85) are saturated for half of our data set and, therefore, were not retained for analysis. P-distances are then computed for amino acid and for nucleotide sequences on the third codon position. To conserve the frame of the coding sequences, the nucleotide alignment is derived from the amino acid alignment (in-house script) of the translated products of the pseudogene and its cognate gene obtained by GENEWISE. The nucleotide alignments between functional paralogs are derived from the amino acid alignments obtained by MUSCLE [35].

### Synteny conservation

Pairwise synteny was examined in windows of five CDS upstream and five CDS downstream of the pseudogene and any of its homolog in another genome. We confirmed conservation of synteny if at least two pairs of homologous neighbors are found within the window.

### Detection of processed pseudogenes

To determine if a pseudogene could arose from a retro-transposition event, we systematically checked for classical retro-transposition hallmarks: lack of intron with respect to their paralog CDS, retrotransposon-related sequences within the first flanking annotated elements of the pseudogene (one upstream and one downstream), and polyA-tail at the 3'-end of the pseudogene. For this last check, we analyzed the A content of the 3'-end flanking region (500 nucleotides) of each pseudogene. We considered as a potential poly(A)-tail, a window of 50 nucleotides containing at least 35 adenines, with at least one stretch of 5 adenines, and less than 10 thymines. To avoid fortuitous signals due to AT-rich sequences, the same procedure was performed on 1000 random sequences (with identical nucleotide composition). Only sequences for which no signal was detected in the random sequences were considered as potential poly(A)-tail.

### Statistical analysis

Correlation tests were done with the method of Spearman implemented in R [36]. We accepted a correlation between data sets when the p-value was lower than 0.05.

## Results

### Strategy

We systematically searched for all potential pseudogenes of ancient protein-coding genes within 8 completely sequenced and annotated genomes of Hemiascomycetes: *Saccharomyces cerevisiae *and *Candida glabrata *(two *Saccharomycetaceae *that underwent ancient polyploidization), *Zygosaccharomyces rouxii*, *Kluyveromyces lactis*, *Kluyveromyces thermotolerans*, *Saccharomyces kluyveri *(four protoploid *Saccharomycetaceae*), and *Debaryomyces hansenii *and *Yarrowia lipolytica*, members of the «CTG» group and of the *Dipodascaceae*, respectively (http://www.genolevures.org). To do this, we compared each genome sequence to the set of 44,174 protein-coding sequences (CDS) annotated in these genomes (see *Methods*). We considered here as pseudogene any sequence that simultaneously -i- does not overlap an already annotated genetic element, -ii- shares sequence similarity with an annotated CDS (either in the same genome or in one or several other yeast genomes considered) and -iii- shows disabling mutations in the reading frame: in-frame stop codon, frameshift mutation or truncation of more than 30% relatively to the CDS. We chose this limit of 30% because, among the functional members of a given protein coding gene family, the length variation does not exceed 30% in the majority of cases (data not shown). Inactive pseudogenes resulting solely from mutations in promoters, as well as pseudogenes without detectable similarity among the eight studied genomes are, therefore, excluded from our analysis. Similarly, we do not question the genome annotations: an annotated gene that appears truncated compared to other homologous sequences will not be considered as a pseudogene because truncation does not necessarily imply inactivation (experimental work is needed to precise such a point).

### Complete set of detected pseudogenes and sequence degradation patterns

We found a total of 871 pseudogenes among the 8 yeast genomes (see Additional file 1, Table S1 for complete list and *Methods *for nomenclature). They correspond to 418 distinct protein families or singletons. The total number of pseudogenes varies significantly between species: from 38 in *C. glabrata *to 230 in *Y. lipolytica*, corresponding to only 0.7% and 3.6% of the annotated CDS, respectively (Table 1).

**Table 1:** Number and types of pseudogenes identified in the eight yeast genomes studied.

Species	Genome size^a^	Total CDS	Pseudogenes^b^	« Full-size »^*c*^(≥ 70%)	3'-Truncation^*d*^ (<70%)	5'-Truncation^e^ (<70%)
*S. cerevisiae*	12.1	5769	77 (1.3)	0.57	0.21	0.22
*C. glabrata*	12.3	5204	38 (0.7)	0.47	0.32	0.21
*Z. rouxii*	9.8	4998	105 (2.1)	0.42	0.28	0.30
*K. thermotolerans*	10.4	5104	68 (1.2)	0.54	0.16	0.29
*S. kluyveri*	11.3	5308	117 (1.3)	0.63	0.19	0.18
*K. lactis*	10.7	5084	61 (2.2)	0.66	0.18	0.16
*D. hansenii*	12.2	6273	175 (2.8)	0.37	0.28	0.35
*Y. lipolytica*	20.5	6434	230 (3.6)	0.36	0.3	0.35

^a^. in Megabases, except rDNA.

^b^. Pseudogenes of protein coding sequences only. The percentage of pseudogenes relative to CDS is indicated in parenthesis.

^c^. Proportion of « full-size » pseudogenes, *i.e*. extending over more than 70% of their bestmatch length.

^d^. Proportion of pseudogenes extensively truncated at their 3'-end.

^e^. Proportion of pseudogenes extensively truncated at their 5'-end.

The pseudogenes identified exhibit a large panel of sequence degradation, ranging from only a few mutational disablements to extensive truncations. We distinguished here the «full-size» pseudogenes, *i.e*. those extending over more than 70% of their closest functional homolog, from the «truncated» pseudogenes (Table 1).

In *S. cerevisiae, S. kluyveri*, *K. thermotolerans *and *K. lactis*, the number of «full-size» pseudogenes exceeds truncated ones. The opposite is true for *C. glabrata*, *Z. rouxii*, *D. hansenii *and *Y. lipolytica*. There are as many pseudogenes truncated from their 3'-end, as those truncated from their 5'-end (Table 1), consistent with the idea that truncation results from deletion at the DNA level rather than incomplete cDNA formation, characteristic of retro-processed pseudogenes. «Full-size» pseudogenes contain more disabling mutations (in-frame stop codons and frameshifts) than truncated pseudogenes (except in *D. hansenii *and *Y. lipolytica*) (Additional file 1, Table S1). We found four pseudogenes (*DEHA2Bp3*, *DEHA2Cp3*, *DEHA2Dp5*, *DEHA2Ep5*) inactivated by insertion of a DNA fragment of mitochondrial origin (NUMTs), as previously described [37].

### Sequence divergence between pseudogenes and their best functional homologs

**Figure 1:**
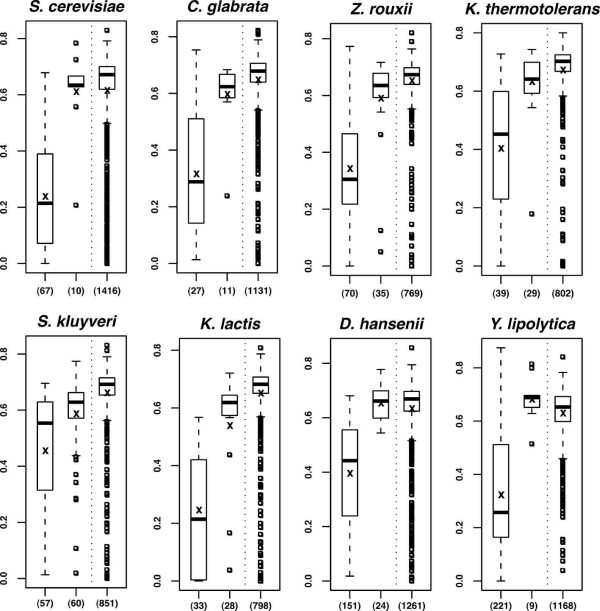
**Boxplot **[67]**of the sequence divergence between pseudogenes and their closest functional homolog**. P-distance (ordinate) is expressed as the fraction of non-identical nucleotides at the third positions of codons (see *Methods*). Left panel: p-distance of pseudogenes whose closest functional homolog (bestmatch) is in the same genome (paralog), central panel: p-distance of pseudogenes whose bestmatch is in another genome, right panel: p-distance of pairs of functional paralogs in the same species. The number of pairs analyzed is indicated in parenthesis (data in Table II).

In the absence of the actual ancestral coding sequences of the pseudogenes, we estimated the distances between pseudogenes and their closest functional homologs (bestmatches) to measure their degree of mutational decay (Additional file 1, Table S1; see *Methods *for details). Distributions of the p-distances were calculated for each yeast species (figure 1). They range from 0 to 0.8 globally. The distributions are different if one considers pseudogenes whose bestmatch is not in the same genome and pseudogenes whose bestmatch is a paralog in the same genome. In the first case, distances are high, consistent with the generally long evolutionary distances between studied species. In the second case, distances are much lower than that observed between functional paralogs in the same genome, suggesting that many pseudogenes arose after recent gene duplication events. The genomes of *S. kluyveri *and *K. thermotolerans *contain the highest proportions of highly diverged pseudogenes (half of them differ by more than 60% from their bestmatches), while the genomes of *S. cerevisiae *and *Y. lipolytica *contain the highest proportions of less diverged pseudogenes (half of them differ by less than 28% from their bestmatches). Note that functional paralogs (right panel) in all these genomes are issued from both ancient (prior to the speciation events) and recent (species-specific) duplications [25].

### Pseudogenes across species, conservation or independent formation

**Figure 2:**
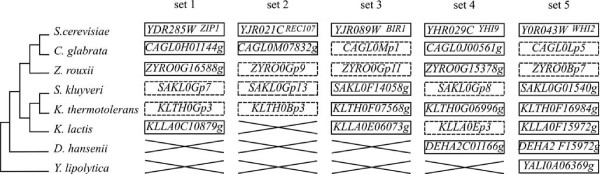
**Conserved pseudogenes at syntenic locations**. Each column represents a set of orthologous sequences in a region of synteny conservation. Vertical dashed lines separate the different regions. Rectangles represent annotated genes, dashed rectangles represent pseudogenes detected in this analysis. All these pseudogenes have no paralog in the genome. The topology of the species phylogeny [68] is given on the left of the figure (branch lengths ignored).

A pseudogene should be free from any functional pressure and, therefore, should not be conserved over long evolutionary periods, unless it acquires a functional role. We, therefore, searched for pseudogenes conserved in several yeast species as a possible functional signature. A total of 274 pseudogenes are shared between at least two species but 263 of them are located outside of any conserved synteny block and probably correspond to independent pseudogenization events of homologous genes in different lineages. The remaining 9 pseudogenes are found among five conserved synteny blocks, defining five groups of orthologous pseudogenes (Figure 2). Note that we did not consider the syntenic orthologs of *YIL009C-A *(*EST3*), *YPL052W *(*OAZ1*) and *YOR239W *(*ABP140*) as pseudogenes, because they all bear the programmed translational frameshift present in the functional genes of *S. cerevisiae *[38]. According to the species phylogeny and following parsimony criterion, three of these sets of orthologous pseudogenes also correspond to independent pseudogenization events of the same gene in distinct lineages (Set 3, 4 and 5 on Figure 2).

Pseudogenes *SAKL0Gp7 *and *KLTH0Gp3*, corresponding to *YDR285W *(*ZIP1*) of *S. cerevisiae *are conserved between *S. kluyveri *and *K. thermotolerans*. *ZIP1 *is a transverse filament protein of the synaptonemal complex that is required for normal levels of meiotic recombination and pairing between homologous chromosomes during meiosis. The 2 orthologous pseudogenes are «full-size» and share a single common frameshift mutation (data not shown). The most parsimonious hypothesis is that the frameshift mutation appeared in the common ancestor of these 2 species.

*REC107 *is a meiotic gene in *S. cerevisiae*, which has been lost several times in hemiascomycetes [39]. This gene is also pseudogenized in three of the newly sequenced genomes studied. *SAKL0Gp13 *and *KLTH0Bp3 *are conserved between *S. kluyveri *and *K. thermotolerans*, suggesting a pseudogenization event in their common ancestor. The orthologous gene is also pseudogenized in *Z. rouxii *(*ZYRO0Gp9*), and probably corresponds to another independent event. Unlike in the previous case, the pseudogenes of this block of conserved synteny are truncated and each copy accumulated several species-specific disabling mutations.

### Conservation of pseudogenes across *S. cerevisiae *strains

The genome sequences for the *S. cerevisiae *non-reference strains are draft assemblies, not suitable for the pseudogene detection procedure developed in this work. However, to get a first estimation of pseudogene conservation among strains, we examined the conservation of the pseudogenes identified in the *S. cerevisiae *reference strain S288C (Additional file 1, Table S1) among 7 other sequenced strains (see *Methods*). We found that 62 of the pseudogenes identified in S288C are also pseudogenized in all other strains, suggesting that the pseudogenization event occurred in their common ancestor. The 14 other pseudogenes of S288C sometimes correspond to intact coding sequences in one or a few other strains, indicating more recent pseudogenization events (Table 2). 11 of these 14 pseudogenes contain at least two degrading mutations (in-frame stop codon or frameshift mutation) and 8 of them are truncated at their 3' or 5' end. The only pseudogene specific to S288C (*SACE0Ip4*) contains only one internal stop codon.

**Table 2:** Pseudogenes in S. cerevisiae S288C with non-degraded homologs in other *S. cerevisiae *strains.

S288C	YJM789	RM11_1A	YPS163	M22	EC1118	JAY291	AWRI1631
*SACE0Ip6*	P	P	p	P	p	intact	Intact
*SACE0Jp5*	P	P	p	P	p	intact	Intact
*SACE0Ap2*	P	P	p	P	p	P	Intact
*SACE0Dp6*	P	P	intact	intact	p	intact	Intact
*SACE0Ip2*	P	P	p	P	intact	intact	Intact
*SACE0Ip4*	Intact	intact	intact	intact	intact	intact	Intact
*SACE0Jp1*	P	P	p	P	intact	intact	Intact
*SACE0Op3*	P	P	intact	P	intact	intact	Intact
*SACE0Op2*	P	P	p	P	intact	intact	P
*SACE0Ap9*	Intact	P	p	P	intact	P	P
*SACE0Op6*	P	P	p	P	intact	P	P
*SACE0Fp3*	P	P	p	P	p	intact	P
*SACE0Ap11*	P	P	p	intact	p	P	P
*SACE0Cp2*	P	P	p	intact	p	P	P
*SACE0Gp1*	P	P	p	intact	p	P	P

p: homologous pseudogene, intact: homolog with non-degraded coding sequence.

### Distribution of the pseudogenes among protein families and corresponding functions

Based on sequence similarity, pseudogenes can be attributed to the functional gene families classified according to their predicted translational products [27]. Such families contain groups of orthologs between species, as well as groups of paralogs resulting from gene duplications and losses (see [40] for a review). In each yeast species, the majority of pseudogenes (from 51 to 88%) belong to gene families with a functionally characterized *S. cerevisiae *member, which can be used to infer the probable function of the gene that was pseudogenized. Interestingly, we observed a bias towards transporters, proteins acting at the periphery of the cell, and enzymes (Table 3). When normalized against the ratios of these functional categories among active genes, the bias is conserved for transporters and proteins acting at the periphery of the cell, but not for enzymes. Among pseudogenes with no ascribed function, there is no over-representation of domains related to these three categories. Although the number of pseudogenes is not directly correlated to the number of its active paralogs in a given family, we observed frequent formation of pseudogenes in these functional categories. For example, there are11 pseudogenes in *D. hansenii *for the sugar transporter family, 12 pseudogenes in *C. glabrata *for the lectin-like protein family, and 25 pseudogenes in *Z. rouxii *for the DUP240 gene family coding for membrane proteins (Additional file 1, Table S2).

**Table 3:** Repartition of the pseudogenes according to the presence/absence of an *S. cerevisiae *homolog, and their functional classification

Species	S.c. homolog with known function	S.c. homolog with unknown function	No S.c. homolog	transporter, periphery of the cell^a^	Enzymes^b^
*S. cerevisiae*	37	31	9	11	15
*C. glabrata*	23	2	13	13	2
*Z. rouxii*	83	9	13	50	14
*K. lactis*	38	6	17	11	15
*K. thermotolerans*	42	6	20	13	11
*S. kluyveri*	78	10	29	33	19
*D. hansenii*	82	7	86	37	24
*Y. lipolytica*	117	6	107	26	38

S.c. is for *S. cerevisiae*.

^a^Number of pseudogenes with a homolog in *S. cerevisiae *coding for proteins involved in transport and/or acting at the periphery of the cell.

^b^Number of pseudogenes without homolog in *S. cerevisiae *coding for enzymes.

A total of 139 pseudogenes are similar to singleton genes, and 288 (one third of total) belong to gene families specific to a given species for which, unless in *S. cerevisiae*, there is usually no functional indication (Additional file 1, Table S2).

### Clustering of the pseudogenes in subtelomeres

Gene densities are nearly constant along yeast chromosomes, except for subtelomeres where the number of active genes is reduced [41], and where genes can be transcriptionally silenced [42]. We, therefore, examined the distribution of the pseudogenes along the chromosomes, separating the subtelomeres (30 kilobases apart from the telomeres or from sequenced chromosome ends) from the central regions (Table 4). In all yeast species, pseudogenes exceed active genes in number (3 to 16 times more) in the subtelomeres. In *S. cerevisiae, C. glabrata *and *Z. rouxii*, more than half of the pseudogenes are found in subtelomeres (70, 60 and 54%, respectively). This proportion is lower in *K. thermotolerans*, *D. hansenii*, *S. kluyveri *and *K. lactis *(47, 40, 36 and 30% respectively), and drops to only 5% in *Y. lipolytica*. The presence of rDNA loci in the subtelomeres of *Y. lipolytica *(6 loci) might prevent some pseudogenes accumulating in these regions, as well as in *D. hansenii *(3 loci) and in *C. glabrata *(2 loci) [25].

**Table 4:** Subtelomeric localization of pseudogenes and presence/absence of annotated paralogs

Species	P. ends^a^	**G. ends**^b^**(%)**	No paralog^c^	Paralog^d^	
				In	Out
S. cerevisiae*	71.4	5.4	3	67	7
*C. glabrata*	60.5	3.2	8	27	3
*Z. rouxii*	54.3	3.2	14	70	21
*K. thermotolerans*	48.5	2	18	39	11
*S. kluyveri*	37.6	2.6	29	57	31
K. lactis*	31.1	2.9	14	33	14
*D. hansenii*	40.0	2.7	8	151	16
*Y. lipolytica*	5.2	1.6	6	221	3

^a^. Percentage of pseudogenes in subtelomeric regions (less than 30 kb from a chromosome end).

^b^. Percentage of active genes in subtelomeric regions.

^c^. Number of pseudogenes without annotated functional paralog in the genome.

^d^. Number of pseudogenes with annotated functional paralog whose closest homolog is in the same genome (in) or in another genome (out).

*. Species for which all chromosomes are fully sequenced, including their telomeric repeats.

### Possible origin of the pseudogenes

**Figure 3:**
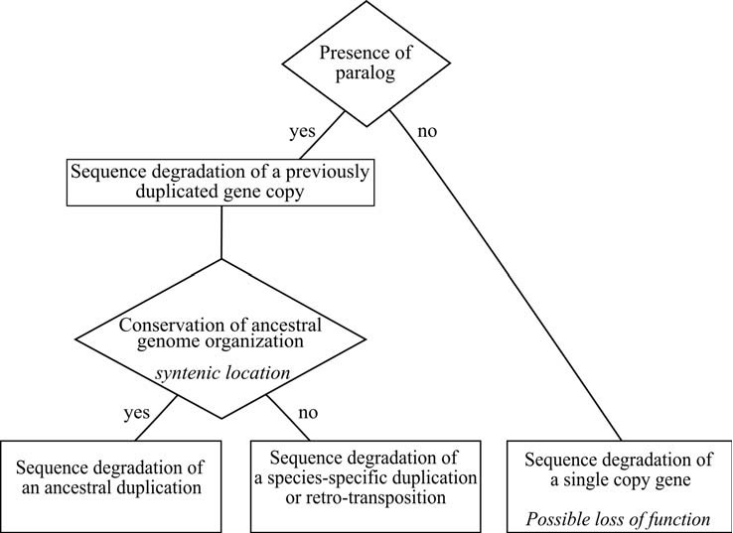
**Possible origin of pseudogenes**. See text for explanations. The diamonds correspond to distinctive criteria and rectangles to deduced origin.

We tried to define the origin of yeast pseudogenes based on the presence or absence of a paralog in the same genome and on the conservation of synteny between species (figure 3). The presence of an active paralog in the genome reveals the occurrence of a previous duplication event, followed by mutational inactivation of one of the 2 copies. The absence of any paralogs reveals the degradation of a single copy gene, hence the loss of the corresponding function in the species. The conservation of synteny is a signature for mutational sequence degradation at the origin of the pseudogene. The non-conservation of synteny is compatible with a species-specific duplication, either a retro-transposition event or a segmental duplication.

#### Pseudogenes originating after species-specific gene duplications

More than half of the pseudogenes arose after a gene duplication event specific to their own genome (Table 5). This corresponds to the vast majority of pseudogenes in *S. cerevisiae*, *D. hansenii *and *Y. lipolytica *(96, 87 and 93%, respectively), *ca*. 70% of the pseudogenes in *C. glabrata *and *Z. rouxii*, and less than 60% in *K. lactis, K. thermotolerans *and *S. kluyveri*. The majority of these pseudogenes probably arose after segmental duplication, but some may be processed pseudogenes, as suggested by a poly(A) tract at their 3'-end or their location next to a retrotransposon-related sequence [43]. This would correspond to only 3.5% of the pseudogenes in *Y. lipolytica*, and 20% of the pseudogenes in *K. thermotolerans*. Only one pseudogene (*DEHA2Ep25*) was identified by the lack of intron compared to its closest functional homolog.

**Table 5:** Classification of pseudogenes according to their possible origin.

Species	Species-specific duplication^a^	Ancestral duplication^b^	Function loss^c^	*duplicated* *pseudos*^d^	*duplicated segment*^e^	*retros*^f^
*S. cerevisiae*	73	1	3	1	0	1, 6
*C. glabrata*	27	3	8	1	1	3, 0
*Z. rouxii*	71	20	14	6	41	1, 3
*K. thermotolerans*	39	11	18	0	17	0, 16
*S. kluyveri*	65	23	29	4	13	1, 12
*K. lactis*	35	12	14	5	7	0, 4
*D. hansenii*	153	14	8	2	62	1, 3
*Y. lipolytica*	213	11	6	6	19	2, 8

^a^. number of pseudogenes originating from mutational inactivation of a duplicated gene copy formed after speciation.

^b^. number of pseudogenes originating from mutational inactivation of a duplicated gene copy formed before speciation.

^c^. number of pseudogenes originating from mutational inactivation of a single copy gene.

^d^. number of duplicated pseudogenes among the first category (^a^).

^e^. number of pseudogenes being part of a duplicated segment involving other adjacent genes among the first category (^a^).

^f^. number of retro-processed pseudogenes, among the first category (^a^), identified by: either the presence of a 3' poly(A)-tail (first number) or the proximity of retrotransposon-related sequence (second number). In each species, the candidates identified by these 2 criteria are different.

**Figure 4:**
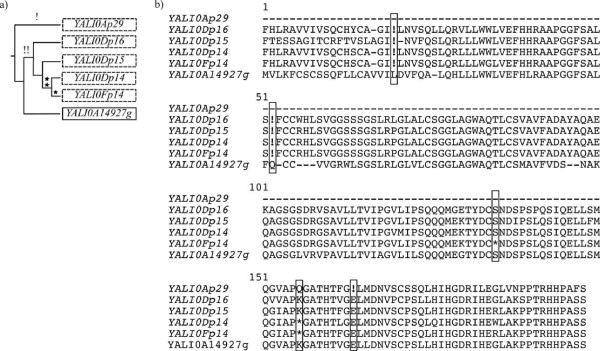
**Scenario for the multiplication of pseudogenes in *Y. lipolytica***. a) Rectangle represents the functional gene, dashed rectangles represent its corresponding pseudogenes. The tree topology is obtained by maximum likelihood reconstruction [69] based on the aligned nucleic acid sequences (branch lengths ignored). The emergence of frameshift mutations (!) and in-frame stop-codons (*) are indicated above corresponding branches. b) Alignment of the translation products of *YALI0A14927g *and its pseudogenes obtained by MUSCLE. frameshift mutations (!) and in-frame stop-codons (*) are boxed.

Interestingly, we found duplications of pseudogenes, as evidenced by their common pattern of degrading mutations. For example, in *Y. lipolytica*, we found five pseudogenes similar to *YALI0A14927g *(Figure 4), which, based on phylogenetic analysis, suggest two original pseudogenization events in the history of this family. Note that these pseudogenes are not located in subtelomeric regions and are not part of larger duplicated regions including other genes. These pseudogenes have thus not been maintained in the genome by some selective pressure on the duplication of adjacent genes. Similarly, two such cases of pseudogene multiplication were encountered in *K. lactis*, as well as 12 cases of pseudogene duplication (Additional file 1, Table S1).

Most pseudogenes located at the chromosome ends arose after a species-specific gene duplication (Table 6): from 60% in *K. thermotolerans *to 100% in *Y. lipolytica*.

**Table 6:** Number subtelomeric pseudogenes according to their possible origin.

Species	Species-specific^a^	Ancestral	Function loss
*S. cerevisiae*	49	1	3
*C. glabrata*	18	0	5
*Z. rouxii*	45	7	5
*K. thermotolerans*	32	8	6
*S. kluyveri*	20	7	4
*K. lactis*	16	2	1
*D. hansenii*	67	4	0
*Y. lipolytica*	11	0	0

See Table 5 for legend.

#### Pseudogenes originating from ancestral gene duplications

**Figure 5:**
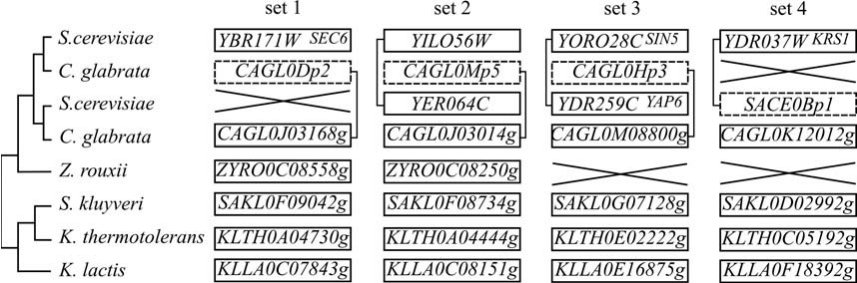
**Pseudogenes in pairs of ohnologs in *Saccharomycetaceae***. Same legend as Figure 2. Pairs of ohnologs, *i.e*. paralogs originating from the whole-genome duplication [70], are linked by brackets.

Pseudogenes formed after ancestral gene duplications are found in all yeast genomes (Table 5). Four pseudogenes, *SACE0Bp1*, *CAGL0Dp2*, *CAGLOHp3 *and *CAGL0Mp5 *were formed after the whole-genome duplication event that occurred in the common ancestor of *C. glabrata *and *S. cerevisiae *[44] (Figure 5). This is a very small number compared to the extensive loss of duplicated genes that occurred by deletion [45]. Strikingly, there are more pseudogenes originated from other ancestral duplications in protoploid *Saccharomycetaceae *and in *D. hansenii *and *Y. lipolytica*, suggesting that selective pressure on duplicated genes are different after whole-genome duplication and other duplication events, such as segmental duplications.

#### Pseudogenes originating without previous gene duplication and putative loss of function

Pseudogenes with no functional paralog (Additional file 2, Table S3) are found in all the studied genomes and could, therefore, correspond to a functional loss. The genomes of *K. lactis*, *K. thermotolerans*, *S. kluyveri *and *C. glabrata *contain the highest proportions of such pseudogenes (23%, 26%, 25% and 20%, respectively). The lowest proportions are found in *D. hansenii *and in *Y. lipolytica *(5% and 3%, respectively), consistent with the higher gene redundancy in these genomes [26]. There is no bias towards any functional category (Additional file 2, Table S3). Pseudogenes homologous to essential genes in *S. cerevisiae *are found in all species, except in *K. lactis*. Repeated pseudogenization of the same gene is also encountered in this category. For example, pseudogenes *CAGL0Mp1 *and *ZYRO0Gp11*, similar to the essential chromosomal passenger gene *YJR089W *(*BIR1*), have no homolog in their respective genome.

Finally, we noticed three interesting pseudogenes in *S. cerevisiae *that correspond to horizontally acquired genes. *SACE0Fp3 and SACE0Fp2 *have no homolog in *S. cerevisiae *but were identified by similarity to *DEHA2D01122g *(similar to a bacterial tryptophan synthase) and *DEHA2E07282g *(similar to a bacterial glyoxalase), respectively. Absence of these genes among hemiascomycetes suggests that four independent horizontal gene transfer events have occurred, the two genes in *S. cerevisiae *being secondarily pseudogenized. The third one, *SACE0Np2 *is a duplicated and identical copy of *SACE0Fp2*, revealing the expansion of horizontally transferred inactivated genes. The transfers must have occurred in an ancestor of all the studied strains of *S. cerevisiae *because the homologs of *SACE0Fp3, SACE0Fp2 *and *SACE0Np2 *are pseudogenized in the 7 other strains examined.

## Discussion

We present here the first systematic search for pseudogenes in the genomes of eight distinct yeast species spanning the whole evolutionary spectrum of hemiascomycetes. Because our method of detection relies on sequence similarity with annotated protein coding genes, the total number of pseudogenes identified (871) represents a minimal estimate (pseudogenes without any functional homolog or with alteration of their promoter sequences were not examined). Among the genomes analyzed here, only *S. cerevisiae *has been the subject of previous systematic analysis of the pseudogene content [6,7]. Our analysis revealed a smaller number of pseudogenes in this species (77) because we deliberately ignored partial overlaps with annotated genetic elements. On the contrary, additional pseudogenes were identified based on our multi-species sequence comparisons. These differences illustrate the difficulties in listing all pseudogenes in a given genome. Despite this fact and numerical variations between yeast species, our results demonstrate that the proportion of pseudogenes compared to active genes remains low in all hemiascomycetous genomes studied (comparable to the proportion of pseudogenes in *D. melanogaster *and much smaller than the proportions in mammalian genomes, see http://pseudogenes.org).

Our comparative analysis of pseudogenes across an entire yeast phylum provides a unique data set to examine their origin and evolutionary conservation. We found that pseudogenes in yeasts are formed either by disabling mutations (in-frame stop codons and frameshift mutations) or by extensive truncations. The general absence of conservation of pseudogenes between yeast species is consistent with their large evolutionary distances [25,26] and indicates that new pseudogenes were formed within each lineage. However, intra-species conservation of pseudogenes is high: about 80% of the pseudogenes in the *S. cerevisiae *reference strain are old enough to be conserved among 7 other strains of this species. Poor conservation of pseudogenes is also observed between mammalian species [46,47]. Most of the pseudogenes correspond to duplicated gene copies, illustrating the extensive dynamics of gene duplications in the yeast genomes, most probably segmental duplications, as observed in *S. cerevisiae *[48]. Only few pseudogenes correspond to duplicated gene copies formed by the ancestral whole-genome duplication common to *C. glabrata *and *S. cerevisiae *[44]. This is consistent with the idea that most duplicated copies were lost by complete deletion, as previously proposed [45,49], or with the possibility that pseudogenes have been degraded beyond recognition, given the time elapsed since the whole-genome duplication event. Among the protoploid *Saccharomycetaceae*, the presence of species-specific pseudogenes within conserved synteny blocks indicates their relatively recent formation, to the notable exception of two ancestral pseudogenes conserved in *S. kluyveri *and *K. thermotolerans*, the more closely related pairs of species studied. The general absence of conserved pseudogenes confirms that most of the observed pseudogenes are on their way to complete degradation.

According to Doniger et al., [32] 49 pseudogenes containing an internal in-frame stop codon (based on our own criteria) and 5 pseudogenes containing a frameshift mutation are present in either M22 or YPS163, while they correspond to intact coding sequences in S288C. From the alignment of the three genomes within coding regions, it appears that the loss of a gene by deletion is much more frequent (3 deleted genes for 19 large indels (>100 bp)) than the creation of a pseudogene by a substitution event (49 pseudogenes for 46807 SNP) or by an indel event (5 pseudogenes for 960 small indels (< 100 bp)). According to Lynch et al. [50], the rate of gene loss, 2.1 × 10^-6 ^per gene per cell division, is much higher than the base-substitutional rate, 0.33 × 10^-9 ^per site per cell division. It should then be much more frequent to lose a gene by deletion than to create a pseudogene.

Unlike their abundance in mammalian genomes [15,51], only few pseudogenes may have originated from retro-transposition events in yeasts. But their presence suggests that, as experimentally demonstrated in *S. cerevisiae *[43,52,53], retro-transposition occurred in these genomes, although a very small number of active retrotransposons are usually present [25,54]. The number of retro-processed pseudogenes in yeasts is, however, probably underestimated because their identification is difficult given the small number of intron-containing genes. The few detected pseudogenes homologous to intron-containing genes are all truncated and do not span the intron insertion-site, except in one case (*DEHA2Ep25*).

Pseudogenes without functional paralog suggest functional loss in the corresponding species, unless a non-homologous gene encodes a similar function [55]. In *S. cerevisiae*, the fact that about half of the pseudogenes correspond to unknown functions is striking given that there remain only 17% of the genes not yet functionally characterized in this species [56]. This suggests that the divergence of functional repertoire between yeast species primarily concerns functions not yet identified. The frequent occurrence of pseudogenes corresponding to transporters or protein acting at the periphery of the cell may be correlated with the tendency of such genes to cluster in the subtelomeric regions, which are highly dynamic in terms of gene duplications and losses [38,41,57] and often concern functions involved in the adaptation of the species to its environment [22,58]. The highly dynamical behavior of such families is supported by the fact that 3 to 16 times more pseudogenes than genes are found in the subtelomeric regions. The vast majority of pseudogenes in the subtelomeres originated from a species-specific duplication. This bias could be correlated to the accelerated base-pair substitution observed in the subtelomeres, which probably also contributes to adaptive evolution [59]. It also suggests that pseudogenes issued from ancestral duplication and function loss are not preferentially maintained in subtelomeres with respect to central regions of chromosomes.

In *S. cerevisiae*, pseudogenes of highly connected genes are significantly under-represented, possibly reflecting the lower propensity of gene loss among these genes: 6% of the pseudogenes of genes coding for protein in complexes against 28% of the active genes [60] and 59% of pseudogenes of genes with a genetic interaction profile against 75% of the active genes [61].

Whether pseudogenes correspond to intermediate gene states before complete erasure or steady states conferring selective advantages remains an open question and may be case-specific. Our data provide no indication as to whether pseudogenes may be transcribed or not. However, according to available experimental data in *S. cerevisiae *(oligonucleotide tiling array experiments [62], 3'-long SAGE approach [63], and direct RNA sequencing [64]), 12 of the 77 detected pseudogenes appear to be transcribed (Table 7). Evidence of transcription also exists in *C. glabrata *and *Y. lipolytica *(H. Müller, personal communication; C. Neuvéglise, personal communication). The transcription products of pseudogenes may be directly targeted to the degradation machinery such as NMD [65], but they could also play some role in the cell by interfering with the expression of functional genes. Moreover, as previously suggested [6], pseudogenes with few disabling mutations could constitute a reservoir of functional protein products if recoding events occur, such as programmed frameshift (See [38] for a comparative analysis of the programmed frameshifting in *Saccharomycetales*), or bypass of the stop-codon [66].

**Table 7:** Pseudogenes with evidence of transcriptionin *S. cerevisiae*

Name	stops	frameshifts	R.L	Reference
SACE0Ap1	0	1	1	[63]
SACE0Ap2	1	0	0.9	[63]
SACE0Ap7	1	4	0.11	[63]
SACE0Ap13	0	1	1	[63]
SACE0Bp1	5	15	1	[62-64]
SACE0Cp2	2	0	0.06	[63]
SACE0Cp3	10	7	1	[63]
SACE0Dp6	1	0	0.82	[62,63]
SACE0Dp7	5	11	0.57	[63]
SACE0Hp1	1	8	0.86	[62]
SACE0Lp4	0	0	0.55	[64]
SACE0Pp4	1	2	0.77	[62]

The number of disabling mutations within each pseudogene is given in columns 2 and 3. The relative length (R.L) of the pseudogene with respect to its closest functional homolog is indicated in column 4. Last column indicates the reference of the data set where evidence of transcription is found (see text for details). For a given chromosome, all identified pseudogenes are separated by several genes. There is no bias of any kind among these pseudogenes.

## Conclusions

Pseudogenes are found in all yeast genomes, albeit in limited number compared to genomes of multi-cellular eukaryotes. They mostly result from lineage-specific mutational degradations that may correlate with species adaptation to their environment. Yeast pseudogenes show a wide range of mutational alterations, consistent with their rapid evolution, hence their absence of conservation between species. Along with complete gene deletion, pseudogene formation contributes to the rapid genome evolution by gene duplication and loss in yeasts. The paucity of observed pseudogenes across the entire phylum of Hemiascomycetes suggests that pseudogene formation is not the main mechanism of gene loss within these genomes. This could be explained by the low estimated rate for pseudogene formation across *S. cerevisiae *strains, compared to the estimated rate of gene deletion. However, despite their unlikely occurrence, pseudogenes do exist in the yeast genomes. They appear mainly by species-specific duplications and testify for the adaptation of the cell to its environment. Their poor conservation across species suggests that most of them are likely to disappear.

## Authors' contributions

IL designed and conducted the analysis, implemented the algorithms and wrote the manuscript. BD contributed to the interpretation of the results and to the writing of the manuscript. Both authors read and approved the final manuscript.

## Supplementary Material

Additional file 1 **Table S1 and Table S2**. - Table S1. List and characteristics of the detected pseudogenes in the 8 studied yeast genomes. - Table S2. List of gene families (or singletons) with detected pseudogenes. Click here for file

Additional file 2 **Table S3 and descriptions of Tables S1 and S2**. Descriptions of Tables S1 and S2. - Table S3. Lost functions among the eight studied species. A list of the pseudogenes with no paralog in the different genomes Click here for file
