# Supplementary material for: Origin and fate of pseudogenes in Hemiascomycetes: a comparative analysis
Source: BMC Genomics. 2010 Apr 22;11:260. doi: 10.1186/1471-2164-11-260 (PMC2876123; doi:10.1186/1471-2164-11-260)
Supplement: Additional file 2 — Table S3 and descriptions of Tables S1 and S2. Descriptions of Tables S1 and S2. - Table S3. Lost functions among the eight studied species. A list of the pseudogenes with no paralog in the different genomes [file 1471-2164-11-260-S2.PDF]

Description of Table S1. **List and characteristics of the detected pseudogenes in the 8 studied yeast genomes**.
Description of Table S2. **List of gene families (or singletons) with detected pseudogenes.**
Table S3. **Lost functions among the eight studied species**. A list of the pseudogenes with no paralog in the different genomes

Table S1. **List and characteristics of the detected pseudogenes in the 8 studied yeast genomes**.

The table is in Additional file 2

Pseudogenes are named according to their position along the chromosome (from left to right end, see text for nomenclature). Chromosomes, coordinates and orientation of pseudogenes are given in columns 2-5. The closest functional homolog among the 8 species studied (bestmatch) and classification of its product into protein families are given in column 6-7. Gene nomenclature refers to (16), family classification refers to (58).

Columns 8-10: characteristics of pseudogene by comparison to its bestmatch.

- R.L: relative length of the pseudogene with respect to its bestmatch length.
- D.A: amino acid divergence between the pseudogene and its bestmatch (see METHODS).
- D.N: nucleotide divergence between the pseudogene and its bestmatch (see METHODS).

Columns 11-12:

- Stop: number of in-frame stop codons according to FASTY
- FS: number of frameshifts according to FASTY

Column 13: pseudogene(s) of the same family, with the same pattern of disabling mutations. Pseudogenes with an asterisk are part of a larger duplicated region including other genes.

Note that a few pseudogenes were already mentioned in the original annotation of *C. glabrata*, *D. hansenii*, *K. lactis* and *Y. lipolytica* (13), and the result of the present work served for the recent updates of their annotation, as well as for the annotation of the newly sequenced genomes of *Z. rouxii*, *S. kluyveri* and *K. thermotolerans* (24).

Table S2. **List of gene families (or singletons) with detected pseudogenes.**

The table is in Additional file 2

The phyletic patterns of each family indicate the presence (s, c, r, l, t, k, d, y) or absence (-) of the pseudogene members (column 2) and of the active gene members (column 4) in each species. The one-letter abreviations are as follows : ‘s’ for *S. cerevisiae*, ‘c’ for *C. glabrata*, ‘r’ for *Z. rouxii*, ‘l’ for *K. lactis*, ‘t’ for *K. thermotolerans*, ‘k’ for *S. kluyveri*, ‘d’ for *D. hansenii* and ‘y’ for *Y. lipolytica*.
The general annotation of the *S. cerevisiae* gene members is given in column 7, according to the annotation of the gene indicated in column 6.
The number of pseudogenes is not correlated to the number of their functional paralogs in the same family (from 1 to at least 20 active paralogs can be found per pseudogene). In addition, 61 pseudogenes have been identified by similarity with genes from another species, because they have no paralog and are unique in their genome.

Table S3. **Lost functions among the eight studied species.**

Name[a]	*Functional homolog*	*Annotated function in S. cerevisiae or annotation by similarity*
SACE0Fp3	*DEHA2D01122g*	weakly similar to Q899U5 *Clostridium tetani* Threonine dehydratase
SACE0Fp2	*DEHA2E07282g*	similar to Q9I565 *Pseudomonas aeruginosa* PA0880 Probable ring-cleaving dioxygenase
SACE0Np2	*DEHA2E07282g*	similar to Q9I565 *Pseudomonas aeruginosa* PA0880 Probable ring-cleaving dioxygenase
CAGL0Lp4	*YCR020W-B HTL1*	Component of the RSC chromatin remodeling complex
CAGL0Lp5	*YOR043W WHI2*	Protein required with binding partner Psr1p for full activation of the general stress response
CAGL0Mp1	*YJR089W BIR1*	Essential chromosomal passenger protein involved in coordinating cell cycle events
ZYRO0Bp8	*YDR277C*	Protein involved in control of glucose-regulated gene expression
ZYRO0Bp7	*YOR043W WHI2*	Protein required with binding partner Psr1p for full activation of the general stress response
ZYRO0Dp8	*YHR084W STE12*	Transcription factor, activates genes involved in mating or

		pseudohyphal/invasive growth pathways
ZYRO0Gp9	*YJR021C REC107*	Protein involved in early stages of meiotic recombination
ZYRO0Gp11	*YJR089W BIR1*	Essential chromosomal passenger protein involved in coordinating cell cycle events
KLTH0Ap7	*YCR054C CTR86*	Essential protein of unknown function
KLTH0Bp3	*YJR021C REC107*	Protein involved in early stages of meiotic recombination
KLTH0Cp5	*YGL256W ADH4*	Alcohol dehydrogenase isoenzyme type IV
KLTH0Dp6	*YDL185W TFP1*	Vacuolar ATPase V1 domain subunit A containing the catalytic nucleotide binding sites
KLTH0Ep7	*YGL256W ADH4*	Alcohol dehydrogenase isoenzyme type IV
KLTH0Ep9	*YNR064C*	Epoxide hydrolase member of the alpha/beta hydrolase fold family
KLTH0Fp3	*YLL057C JLP1*	Fe(II)-dependent sulfonate/alpha-ketoglutarate dioxygenase involved in sulfonate catabolism
KLTH0Gp3	*YDR285W ZIP1*	Transverse filament protein of the synaptonemal complex

KLTH0Hp8	*YALI0C10311g*	similar to uniprot|P50505 *Debaryomyces occidentalis* HAK1 high affinity potassium transporter
SAKL0Dp4	*YBR272C HSM3*	Proteasome-interacting protein regulatory particle (RP)
SAKL0Ep17	*YDL103C QRI1*	Essential UDP-N-acetylglucosamine pyrophosphorylase
SAKL0Hp7	*YDL200C MGT1*	DNA repair methyltransferase (6-O-methylguanine-DNA methylase)
SAKL0Gp7	*YDR285W ZIP1*	Transverse filament protein of the synaptonemal complex
SAKL0Cp4	*YDR499W LCD1*	Essential protein required for the DNA integrity checkpoint pathways
SAKL0Bp7	*YFL023W BUD27*	Protein involved in bud-site selection nutrient signaling and gene expression
SAKL0Cp9	*YJL168C SET2*	Histone methyltransferase with a role in transcriptional elongation
SAKL0Gp13	*YJR021C REC107*	Protein involved in early stages of meiotic recombination
SAKL0Dp8	*YML051W GAL80*	Transcriptional regulator involved in the repression of GAL genes in the absence of galactose
SAKL0Bp12	*YNR064C*	Epoxide hydrolase member of the alpha/beta hydrolase fold family

SAKL0Fp2	*YNR064C*	Epoxide hydrolase member of the alpha/beta hydrolase fold family
DEHA2Bp9	*YDR280W RRP45*	Protein involved in rRNA processing
DEHA2Dp22	*YJL085W EXO70*	Essential 70kDa subunit of the exocyst complex
DEHA2Ep24	*YGL040C HEM2*	Delta-aminolevulinate dehydratase
DEHA2Ep15	*YDR127W ARO1*	Pentafunctional arom protein catalyzes steps 2 through 6 in the biosynthesis of chorismate
DEHA2Ep13	*YALI0E11473g*	similar to uniprot|P50505 *Debaryomyces occidentalis* HAK1 high affinity potassium transporter
DEHA2Gp11	*YOL081W IRA2*	GTPase-activating protein that negatively regulates RAS
YALI0Cp8	*YOR188W MSB1*	Protein involved in positive regulation of both 1,3-beta-glucan synthesis and the Pkc1p-MAPK pathway
YALI0Dp22	*YOR274W MOD5*	Delta 2-isopentenyl pyrophosphate:tRNA isopentenyl transferase
YALI0Dp8	*YHR201C PPX1*	Exopolyphosphatase

YALI0Fp32	*YKL038W RGT1*	Glucose-responsive transcription factor
YALI0Fp31	*YGR112W SHY1*	Mitochondrial inner membrane protein required for normal respiration
YALI0Fp5	*YGL190C CDC55*	Non-essential regulatory subunit B of protein phosphatase 2A

[a]. pseudogenes without functional paralog (pseudogenes similar to genes with uncharacterized function are not listed).
